# Increased Renal Clearance of Rocuronium Compensates for Chronic Loss of Bile Excretion, via upregulation of Oatp2

**DOI:** 10.1038/srep40438

**Published:** 2017-01-13

**Authors:** Long Wang, Mai-Tao Zhou, Cai-Yang Chen, Wen Yin, Da-Xiang Wen, Chi-Wai Cheung, Li-Qun Yang, Wei-Feng Yu

**Affiliations:** 1 Department of Anaesthesiology, Eastern Hepatobiliary Surgery Hospital, the Second Military Medical University, 225 Changhai Road, Shanghai, China; 2 Department of Anaesthesiology, 101th Hospital of Chinese People’s Liberation Army, 101 North Xingyuan Road, Wuxi, Jiangsu, China; 3 Department of Anaesthesiology, Ren Ji Hospital, School of Medicine, Shanghai Jiao Tong University, 160 Pudian Road, Shanghai, China; 4 Department of Anaesthesiology, Queen Mary Hospital, The University of Hong Kong, 102 Pokfulam Road, Hong Kong, China

## Abstract

Requirement for rocuronium upon surgery changes only minimally in patients with end-stage liver diseases. Our study consisted of both human and rat studies to explore the reason. The reduction rate of rocuronium infusion required to maintain neuromuscular blockade during the anhepatic phase (relative to paleohepatic phase) was examined in 16 children with congenital biliary atresia receiving orthotopic liver transplantation. Pharmacodynamics and pharmacokinetics of rocuronium were studied based on BDL rats. The role of increased Oatp2 and decrease Oatp1 expressions in renal compensation were explored. The reduction of rocuronium requirements significantly decreased in obstructively jaundiced children (24 ± 9 vs. 39 ± 11%). TOF50 in BDL rats was increased by functional removal of the kidneys but not the liver, and the percentage of rocuronium excretion through urine increased (20.3 ± 6.9 vs. 8.6 ± 1.8%), while that decreased through bile in 28d-BDL compared with control group. However, this enhanced renal secretion for rocuronium was eliminated by Oatp2 knock-down, rather than Oatp1 overexpression (28-d BDL vs. Oatp1-ShRNA or Oatp2-ShRNA, 20.3 ± 6.9 vs. 17.0 ± 6.6 or 9.3 ± 3.2%). Upon chronic/sub-chronic loss of bile excretion, rocuronium clearance via the kidneys is enhanced, by Oatp2 up-regulation.

Rocuronium is steroidal neuromuscular blocking agent with a rapid onset and intermediate duration of action^1^. Rocuronium is eliminated primarily as unchanged molecule in the bile, with only minor contribution of urinary elimination^2^,^3^,^4^.

However, two previous human studies reported that rocuronium infusion requirement was not significantly reduced in the anhepatic phase of liver transplantation^5^,^6^. Also, plasma clearance of rocuronium was not influenced by end-stage liver diseases^7^,^8^. Based on these observations, extra-hepatic clearance for rocuronium is reasonable to be inferred. Obviously, the kidneys are the most likely candidate for such alternative pathway^6^.

Organic anion transporting polypeptides (rodents: Oatps; human: OATPs) are a superfamily of transmembrane transporters that eliminates a wide variety of structurally unrelated amphipathic organic compounds, including rocuronium, bile salts and thyroid hormones^9^,^10^,^11^,^12^. Impaired bile secretion, such as upon cholestasis, alters the expression of Oatps in the kidneys and cholangiocytes^13^,^14^. We therefore examined the expression of Oatps in the liver and kidneys upon bile duct ligation (BDL) in the current study.

The current study consisted of a human study which examined the requirement of rocuronium to maintain neuromuscular blockade in anhepatic phase of orthotopic liver transplantation (OLT), and rat studies to determine the possible contribution of the kidneys to rocuronium clearance under the condition of impaired bile secretion. In human study, the reduction of rocuronium infusion requirement during the anhepatic phase (relative to paleohepatic phase) of OLT was examined in 16 children with congenital biliary atresia. The rat experiments were conducted on 7 or 28 days after ligating the bile duct. Potential contribution of the renal excretion vs. hepatic secretion on rocuronium clearance was examined by functionally removing the liver vs. kidneys (pharmacodynamics) and by concentration determination (pharmacokinetics). The underlying mechanisms focused on Oatp1 and Oatp2 in kidneys were explored by Oatp2 knock-down and Oatp1 overexpression via adeno-associated virus (AAV).

## Results

### Human studies

Sixteen children were included in this study. Demographic and clinical information including age, gender, weight, total bilirubin, serum albumin, PT and ascites, whether undergoing Kasai portoenterostomy, CTP score, was shown in Table 1

**Figure 1:**
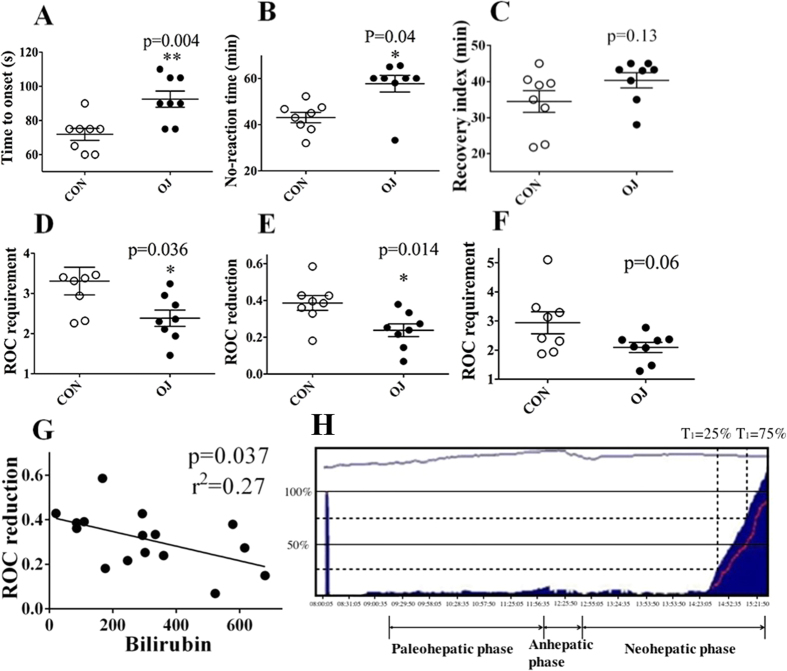
Obstructive jaundice induced extra-hepatic compensation for rocuronium clearance upon compromised bile secretion. (**A**) The onset time of rocuronium after bolus injection. (**B**) No reaction period of TOF (time from T_1_ disappearing to appearing. (**C**) Recovery index (time for T_1_ from 25% to 75%). (**D and F**) the infusion requirement of rocuronium to maintain a stable neuromuscular in the anhepatic (**E**) and neohepatic phase (**F**). (**E)** The reduction of rocuronium requirement in the anhepatic phase (relative to paleohepatic phase), which negatively correlated with the extra-hepatic compensation. (**G**) The negative correlation between the reduction of rocuronium and serum bilirubin. (**H**) Continuous recording of the twitch response of adductor pollicis to TOF stimulation. *Significantly (p < 0.05) different from control. **Significantly (p < 0.01) different from control.

The onset time after rocuronium bolus injection (Fig. 1A) and no reaction period of TOF (time from T_1_ disappearing to appearing, Fig. 1B) in obstructively jaundiced (OJ) children were significantly larger than that in control group (93 ± 13 vs. 72 ± 10 s; 58 ± 10 vs. 43 ± 6.3 min, respectively). The requirement of rocuronium to maintain a stable neuromuscular blockade was significantly lower in obstructively jaundiced group during the anhepatic phase (2.4 ± 0.57 vs. 3.3 ± 0.97 μg•kg^−1^•min^−1^, Fig. 1D), while the reduction of rocuronium requirement during the anhepatic phase (relative to paleohepatic phase), which negatively correlated with the degree of extrahepatic compensation, significantly decreased in OJ group compared with that in control group (24 ± 9 vs. 39 ± 11%, Fig. 1E). The reduction of rocuronium negatively correlated with serum bilirubin (r^2^ = 0.27, p = 0.037, Fig. 1G).

### Rat experiments

**Figure 2:**
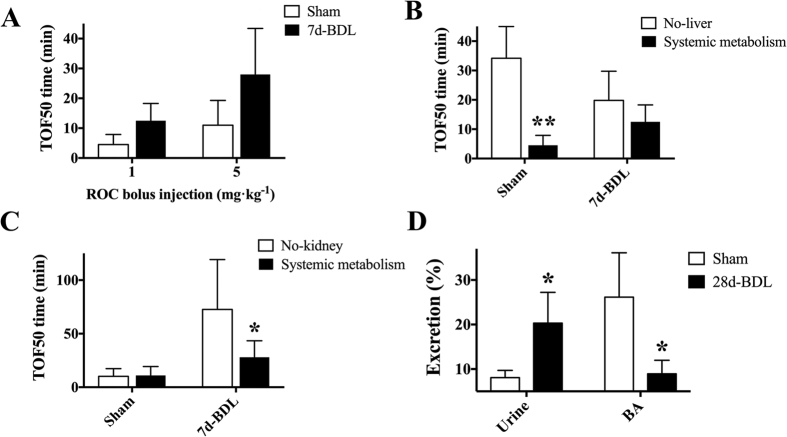
Increased renal clearance of rocuronium compensated for compromised bile secretion in BDL rats. (**A)** The TOF50 time after rocuronium bolus injection (1 or 5 mg·kg^−1^) in Sham and 7d-BDL groups. (**B**) Comparisons of TOF50 time after a single i.v. bolus injection of rocuronium of 1 mg·kg^−1^ in Sham and 7-d BDL rats with or without liver. (**C**) Comparisons of TOF50 time after a single i.v. bolus injection of rocuronium of 5 mg·kg^−1^ in Sham and 7-d BDL rats with or without kidneys. (**D**) Comparisons of excretion percentage through bile and urine between 28-d BDL rats and Sham rats. *Significantly (p < 0.05) different. **Significantly (p < 0.01) different.

7d-BDL increased serum bilirubin (445 ± 163 vs. 155 ± 99μmol·L^−1^, p < 0.01). The majority of elevated bilirubin was the conjugated type. 7d-BDL alone potentially increased TOF50 (11 ± 8.3 vs. 4.5 ± 3.4 and 28 ± 15 vs. 13 ± 5.8 min for 1 and 5 mg•kg^−1^, respectively, Fig. 2A), although there was no significant difference. Removing the liver in 7d-BDL rats did not significantly affect TOF50 (no-liver vs. systemic metabollism, 13 ± 5.8 vs. 20 ± 9.9 min; p > 0.05; Fig. 2B). Removing the kidneys in 7d-BDL rats increased TOF50 (no-kidney vs. systemic metabolism, 73 ± 47 vs. 28 ± 15 min; p < 0.05; Fig. 2C).

After injection of rocuronium 5 mg·kg^−1^, The excretion percentage of the administered dose through the urine statistically increased in 28d-BDL group compared with control group in the first 4 hours after injection (20.3 ± 6.9 vs. 8.6 ± 1.8%, p < 0.05; Fig. 2D), while opposite result was observed in bile excretion (8.9 ± 3.0 vs. 28.4 ± 9.3%, p < 0.05; Fig. 2D).

**Figure 3:**
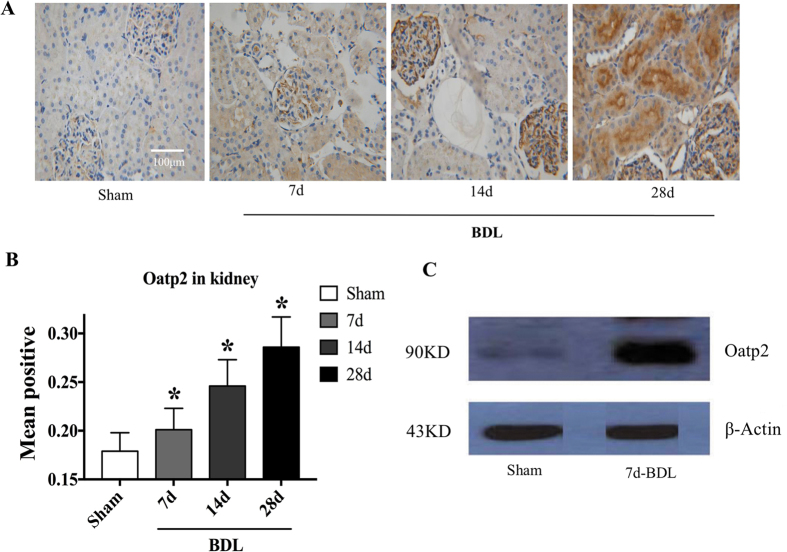
Renal expression of Oatp2 was up-regulated by BDL. (**A**) represents expression of Oatp2 in Sham, 7d, 14d and 28d-BDL rats detected by IHC. (**B**) Represents statistical analysis of positive Oatp2 in the four groups. (**C**) Western blot of Oatp2 in kidney plasma membranes from Sham (lane 1) and 7-d BDL (lane 2) rats. The image has been cropped and 73KD represents Oatp2. *Significantly (p < 0.05) different from Sham. KD = kilodaltons.

**Figure 4:**
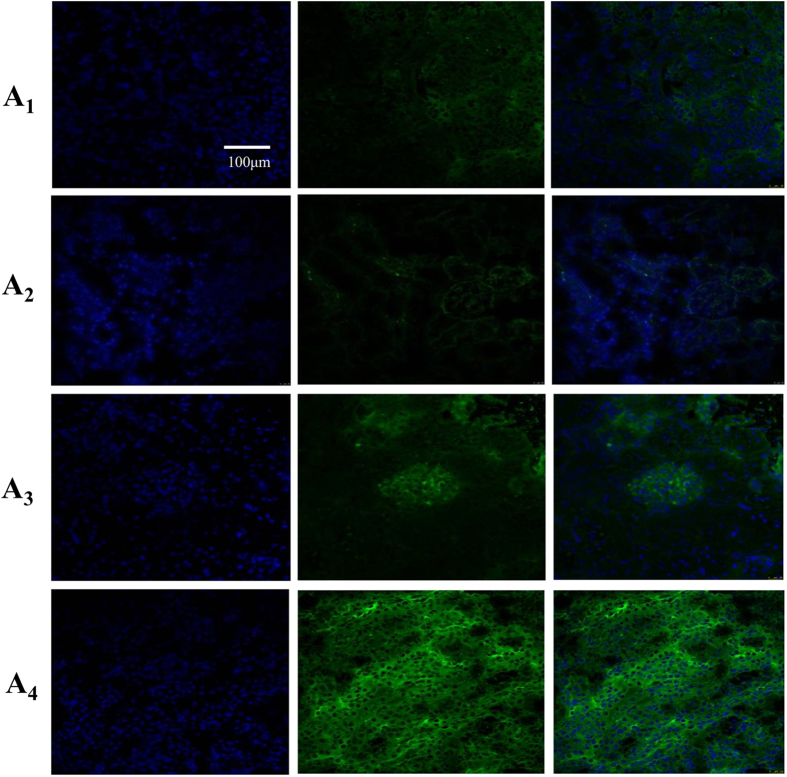
Immunofluorescent for renal expression of oatp2 from Sham and BDL rats: A_1_ Sham rats; A_2_ 7d-BDL rats; A_3_ 14d-BDL rats; A_4_ 28d-BDL rats. DAPI appears as a blue signal. Antibodies to Oatp2 were labeled with fluorescein isothiocyanate (FITC), which appears as a green signal.

Previous studies have reported that the excretion of rocuronium is mediated by Oatp1, Oatp2 and Oatp3 in rodents^15^,^16^,^17^. Considering the evidence that the oatp3 mRNA is undetectable in kidneys^18^, we mainly focused on the Oatp1 and Oatp2 in kidneys. BDL increased Oatp2, mostly in the glomerulus on day 14 after the surgery and throughout the entire nephronay on day 28 (Fig. 3A and 4). The mean positivity for Otap2 in kidney was increased to 20.1 ± 2.2, 24.6 ± 2.7, 28.6 ± 3.1% on 7, 14 and 28 days after the surgery, respectively vs. 17.9 ± 1.9% in Sham group (p < 0.05, Fig. 3B). Western blot analysis also confirmed increased Oatp2 in the kidney of BDL rats (Fig. 3C). Meanwhile, BDL down-regulated the mean positivity of Oatp2 in the liver (18.2 ± 2.1, 17.1 ± 1.9 and 16.9 ± 1.8% on 7, 14 and 28 days after the BDL surgery respectively, vs. 20.0 ± 2.2% prior to BDL, p < 0.05, Figure S1). The mean positivity of Oatp1 in kidney decreased (28.2 ± 3.2, 26.9 ± 2.9 and 25.9 ± 2.8% on 7, 14 and 28 days after the BDL surgery, respectively vs. 29.8 ± 3.4% prior to BDL, p < 0.05, Figure S2).

**Figure 5:**
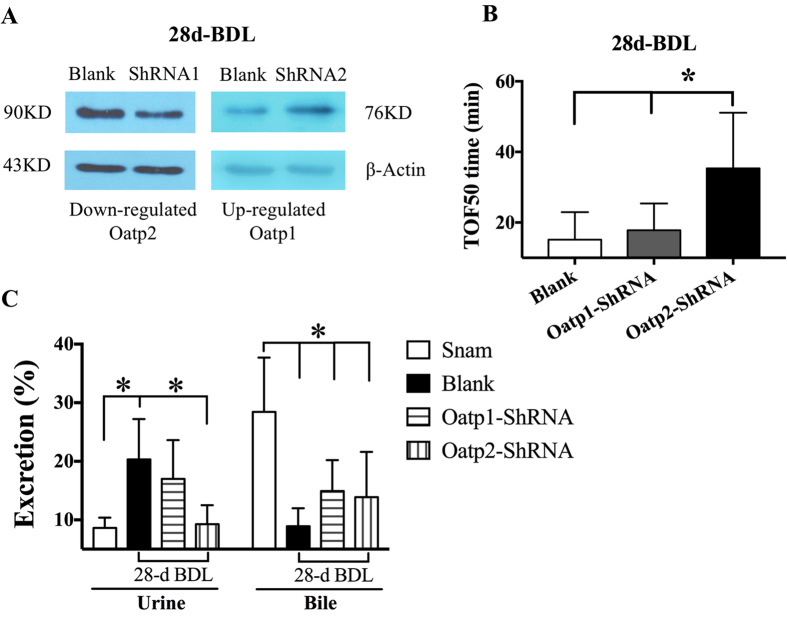
Renal compensation for rocuronium clearance was eliminated by Oatp2 knock-down in kidneys. (**A**) The renal Oatp1 and Oatp2 expressions in 28d-BDL rats administered with blank solution, AAV-rSlco1a1 or rSlco1a2 ShRNA. The image has been cropped and 90KD represents Oatp1 and Oatp2. (**B**) TOF50 time in 28d-BDL rats administered with blank solution, AAV-rSlco1a1 or rSlco1a2 ShRNA. (**C**) Comparisons of excretion percentage through bile acid and urine among Sham and 28-d BDL rats administered with blank solution, AAV-rSlco1a1 or rSlco1a2 ShRNA. *Significantly (p < 0.05) different.

To assess the efficiency of AAV vectors for gene transfer into renal cells, we used H9C2, 293 AAV and HER293 cell line, in which Oatp2 was over-expressed artificially, as an *in vitro* model. Figure S3A demonstrated the AAV vectors efficiently transduced into these cells. Meanwhile, the Oatp2 expression was also significantly inhibited in both mRNA (Figure S3B) and protein level (Figure S3C). The vector solutions were selectively injected into rat kidneys using the procedure as described in Methods. The over-expressed Oatp2 was obviously reversed by AAV vectors in 28d-BDL rats (Figure S3D and 5A). Figure S4 and 5A illustrated that the artificially upregulation of Oatp1 via AAV in kidneys was also successfully established. Oatp2 knock-down rather Oatp1 artificial upregulation in kidneys of 28d-BDL rats significantly prolonged TOF50 (blank vs. Oatp1-ShRNA, or Oatp2-ShRNA, 15 ± 7.8 vs. 18 ± 7.5, or 36 ± 22 min, Fig. 5b), which indicated us the excretion disorder of rocuronium after Oatp2 knock-down. The increased urinary excretion percentage of rocuronium induced by BDL was reversed after Oatp2 knock-down instead of Oatp1 artificial overexpression (28-d BDL vs. Sham, Oatp1-ShRNA or Oatp2-ShRNA, 20.3 ± 6.9 vs. 8.6 ± 1.8, 17.0 ± 6.6 or 9.3 ± 3.2%, Fig. 5C), while Oatp1overexpression and Oatp2 knock-down in kidneys had no effects on bile excretion in BDL rats (28-d BDL vs. Sham, Oatp1-ShRNA or Oatp2-ShRNA, 8.9 ± 3.0 vs. 28.4 ± 9.3, 15.0 ± 5.3 or 13.9 ± 7.7%, Fig. 5C).

## Discussion

The results from the human experiments in the current study demonstrated the reduction of rocuronium requirement during the anhepatic phase (relative to paleohepatic phase) significantly decreased in OJ group compared with that in control group: subjects with OJ prior to the surgery tended to require relatively higher rate of rocuronium infusion in the anhepatic phase. Interestingly, the reduction of the rocuronium negatively correlated with serum bilirubin prior to OLT. In the experiments with BDL rats, TOF50 was increased significantly by removing the kidneys, but not the liver, which was consistent with another interesting finding that BDL induced alternation of excretion pathway for rocuronium (urine increased while bile decreased). Furthermore, the enhanced renal excretion was eliminated by Oatp2 knock-down rather Oatp1 upregulation in kidneys.

The current finding that children with OJ tend to require higher rocuronium infusion rate for muscular blockade in the anhepatic phase of the OLT clearly suggested the existence of compensatory mechanisms for rocuronium clearance upon compromised bile secretion. The results also suggested that the extent of the compensation was dependent on baseline serum bilirubin. Experiments in BDL rats clearly demonstrated that this compensation occurs in the kidneys. In contrast to significantly prolonged TOF50 by kidney removal, removing the blood supply to the liver in 7d-BDL rats did not significantly affect rocuronium clearance, and 28d-BDL rats tended to eliminate rocuronium via urinary excretion rather than bile acid pathway.

Oatps are widely expressed in a variety of organs, including the kidneys, and seem to be responsible for extrahepetic clearance of rocuronium. Combining the evidence that the excretion of rocuronium is mediated by Oatp1, Oatp2 and Oatp3 in rodents^15^,^16^,^17^ and the oatp3 mRNA is undetectable in kidney^18^, we mainly focused on the Oatp1 and Oatp2 in kidney. The altered expressions of Oatp1 and Oatp2 in kidneys and liver are consistent with previous studies^19^,^20^ which might contribute to reduce the accumulation of biliary components. We speculated that the surge of bilirubin or bile acids upon BDL stimulated increased expression of Oatp2 in nephron units. In the kidneys, Oatp2 functions as an efflux transporter upon cholestasis^21^. Combined with our results that Oatp2 knock-down rather Oatp1 overexpression in kidneys eliminated the renal compensation for rocuronium excretion in BDL rats, we could conclude adaptive increase of Oatp2 in kidneys could reduce plasma bile acids and other organic anions, such as exogenously administered rocuronium

Oatp1 in renal tubules reabsorb a number of organic compounds that reached the renal tubles^22^. Oatp2 in the liver represents a high-affinity backup system for removal of certain cholephilic substances from portal blood^23^. Lower expression of Oatp1 in the kidneys and Oatp2 in liver of BDL rats discovered in the current study are consistent with increased renal excretion and decreased hepatic secretion upon hepatic damage, although the Oatp1 has been proved to play no role in renal enhanced excretion of rocuronium in the present study.

Pregnane X receptor (PXR) response elements could be detected on the Oatp2 gene and the Oatp2 was up-regulated in PXR knockout mice^24^,^25^. Bile acids as signaling molecules could activate the nuclear hormone receptors PXR. So, the up-regulation of Oatp2 in kidneys may attribute to the activated PXR. BDL is also associated with inflammation. Serum levels of pro-inflammatory cytokines, such as interleukin-1 (IL-1), interleukin-6 (IL-6), and tumor Necrosis Factor alpha (TNF-α), are elevated in BDL rats^26^,^27^. Recombinant TNF-α, IL-1, and IL-6, are each capable of producing down-regulation of Oatp2 mRNA expression in mice^28^,^29^,^30^. However, the specific cellular and molecular mechanisms responsible for cytokines-related regulation of Oatp2 remains to be determined.

In a few previous studies^5^,^6^, reduction of rocuronium requirement in the anhepatic period (relative to that in the paleohepatic phase) is less prominent than that in the current study. Several possible explanations could account for the difference. First, we did not adopt the commonly used veno-venous bypass during the anhepatic period, since blocking the inferior vena cava could decrease blood flow to the kidneys^6^. As a result, renal clearance of rocuronium might have been reduced. Also, the different results may be caused by different subjects adopted which were children with congenital biliary atresia, other than adults with end-stage liver diseases.

Neither electromyography (EMG) nor mechanomyography was used in the current study. Despite of this limitation, we have been using the acceleration transducer to monitor neuromuscular function in similar study, with consistent results^31^,^32^,^33^,^34^. Specifically, our results correlated well with the results obtained using mechanomyography or EMG^35^. Also, TOF50 time was measured using a bioassay, representing a weakness of the study.

In conclusion, kidneys play a prominent role in the clearance of rocuronium when excretion through the bile is diminished, via increased expression of Oatp2 in kidneys. The results from the current study encourage the use of rocuronium in patients with cholestasis.

## Methods

### Clinical study

#### Patients and Anaesthesia

The protocol was approved by the Institutional Ethics Committee of the Renji Hospital and the Shanghai Jiao Tong University. The methods used in our article have been carried out in accordance with the Code of Ethics of the World Medical Association (Declaration of Helsinki) for experiment involving human. All subjects’ guardians gave written informed consent prior to the study. The study is registered with www.chictr.org.cn, number ChiCTR-OOC-16008707.

The study included 16 children with congenital biliary atresia (10 underwent Kasai portoenterostomy) scheduled to receive OLT under general anaesthesia during a period from July to September 2016. The subjects were divided into two groups according to serum bilirubin (n = 8): obstructively jaundiced group (OJ) and control group. Patients who had one or more of the following conditions were not included: a neuromuscular disease such as one of multiple sclerosis, Huntington’s disease, Creutzfeldt-Jakob disease, myasthenia gravis or polymyositis ect. Chronic kidney diseases and acute renal failure. Anaesthesia was induced with ketamine (5–8 mg•kg^−1^, i.m.) and fentanyl (5 μg•kg^−1^, i.v.). After the induction, rocuronium (Esmeron^®^, 0.6 mg•kg^−1^) was immediately given as an intravenous bolus to facilitate tracheal intubation. Anaesthesia was maintained with propofol and rocuronium infusion. Patients were ventilated mechanically with oxygen and air to maintain end-tidal carbon dioxide in the range 30 to 40 mmHg. Blood potassium was maintained at >3.5 mmol•L^−1^. Blood calcium was maintained at >1.0 mmol•L^−1^. Urine output was measured hourly. During the anhepatic phase, portal vein and hepatic vein were completely blocked, instead of bypassing the veno-venous bypass from the lower limbs and portal vein to the jugular vein.

#### Rocuronium Infusion

Rocuronium infusion was commenced when the twitch response in the adductor pollicis muscle to train-of-four (TOF) stimulation fully recovered from the initial bolus injection (0.6 mg•kg^−1^, as described above). Rocuronium was infused at an initial rate of 0.3 μg•kg^−1^•min^−1^, and adjusted to maintain 85–95% neuromuscular paralysis. The infusion rate must be stable for at least 20 min to be included in the analysis of rocuronium requirement.

#### Neuromuscular function monitoring

The level of neuromuscular paralysis was evaluated by measuring the acceleration of the adductor pollicis twitch response to asupramaximal stimulation (0.2msec duration single stimulus at a frequency of 0.1 Hz) to the ulnar nerve at the wrist, via the TOF-Watch^®^SX.

### Rat experiments

All animal experiments were conducted in accordance with the Institutional Guidelines on the Use of Live Animals for Research, and approved by the Animal Careand Use Committee of the Second Military Medical University.

Adult male Sprague-Dawley (SD) rats (250–350 g) were housed under a 12 h light/12 h dark cycle at 22 °C, with free access to food and water until 8 hours prior to pentobarbital anaesthesia (50 mg•kg^−1^; i.p.) after atropine injection (0.25 mg•kg^−1^). Rats were intubated and ventilated (frequency: 90/minute; pressure: 0.015–0.02 Mpa) using a TKR-200 respirator. The end-tidal P_CO2_ was maintained at 35–45 mmHg. Twenty-one rats received BDL. Briefly, the common bile duct was securely ligated close to the hilus and cut between the two ligatures. A group of rats (n = 18) receiving Sham operation (all the procedures with the exception of BDL) was included as a healthy control.

On day 7 after BDL (17 BDL rats, 15 Sham operation rats), the liver was exposed and the liver artery was ligated. The portal vein was blocked with a vascular clamp at its distal end. The left hepatic lobe was partly excised to expose the portal vein along its inferior margin. A heparinized silicone catheter (diameter: 0.2 cm) was inserted into the portal vein. The other end of the silicone catheter was fixed in the left hepatic vein. The vascular clamp was then opened to allow blood flow to the left hepatic vein from the portal vein. For kidneys removal, both renal arteries were ligated at the pedicles.

The right hindlimb of the rat was immobilized and the sciatic nerve was isolated surgically in the popliteal space. A bipolar platinum electrode was attached to the peripheral portion of the nerve. The distal tendon of the right tibial was isolated from the surrounding tissue and connected to a force transducer (TB-611, Nihon Kohden, Tokyo, Japan) at a resting tension of 30 g. TOF stimulation (TOF-Watch^®^ SX), consisting of four supramaximal pulses (0.2msec duration; 2 Hz), was applied to the sciatic nerve via the electrodes every 15 sec. The T_1_ block was maintained at 95-105% for at least 5 min before rocuronium administration (i.v. bolus injection of rocuronium of 1 mg•kg^−1^ or 5 mg•kg^−1^). Neuromuscular block was monitored by measuring the time needed for TOF resumption to 50% (TOF50).

#### In vivo rSlco1a1 or rSlco1a2 -ShRNA Transduction into the Kidneys

To achieve efficient rAAV-DJ-ZsGreen-rSlco1a1 or rSlco1a1 ShRNA delivery into the kidney *in vivo*, we used a catheter-based gene delivery system in rats. The flexible Solo-Cath catheter (2Fr; Solo-mon Scientific, Plymouth Meeting, Pa., USA) was inserted via the left iliac artery and the abdominal aorta, and the tip was placed beneath the left artery. After the aorta was clamped just above the left renal artery, 1 ml of AAV vector solution was injected after 1-2 ml of saline to wash out blood via the catheter. The left renal vein then was clamped for 10 min. Catheter and clip were removed. Meanwhile the BDL procedures were carried out, and rats were sacrificed on day 28.

Urine, bile acid and serum from 28d-BDL rats (n = 5) and Sham rats (n = 4) were collected 4 h after rocuronium administration (i.v. bolus injection 5 mg•kg^−1^). Concentrations of rocuronium were measured by HPLC-MS (Agilent Technologies, CA, USA; Applied Biosystems Sciex, MA, USA).

#### Immunohistochemistry

Paraffin sections (5μm) were deparaffinized and rehydrated. After incubation with 2% bovineserum albumin in Tris-buffered saline (TBS; pH 7.6) at room temperature for 30 min, sections were incubated overnight at 4 °C with either an anti-oatp1 (OATP21-A, 1:200, ADI, Samford, Connecticut, America) or anti-oatp2 (OATP21-B, 1:200, ADI, Samford, Connecticut, America). After extensive washing, the sections were incubated with a polymer enhancer and a polymerized anti-rabbit or anti-mouse IgG (dilution1:200, Jingmei, Shanghai, China) labeled with horseradish peroxidase. The staining was visualized with a DAB kit (Maixin Biologic Technology, Fujian, China), and appeared as buffy granules in cytoplasm.

#### Immunofluorescence

Tissue sections were prepared as described previously^36^. Nonspecific bindings were blocked with 2% bovine serum albumin (BSA) in TBS pH 7.6 at room temperature for 30 min. Then, the tissue sections were incubated overnight at 4 °C with the anti-Oatps antibody. Washing was then performed at room temperature using TBS pH7.6. And then, the tissues were incubated with a cyber green-conjugated goat anti-rabbit IgG antibody (dilution1:200, Jingmei, Shanghai, China) in 2% BSA-TBS at 1:400 for 3 h. The sections were analyzed using a confocal fluorescencemicroscope (Nikon UFX, Tokyo, Japan).

#### Western blotting analysis

Crude plasma membrane (CPM) was prepared as described previously^37^,^38^, and separated using a 7.5% polyacrylamide gel. The proteins were transferred to a polyvinylidene fluoride (PVDF) membrane, and then blocked with 5% non-fat dry milkin TBST buffer containing 0.05% Tween-20 at room temperature for 1 h. The membrane was then incubated overnight at 4 °C with an anti-oatp2 antibody (OATP21-B, 1:200, ADI, Samford, Connecticut, America). After appropriate washing, the membrane was incubated for 1 h with an anti-rabbit IgG antibody (dilution1:200, Jingmei, Shanghai, China) for detection of Oatp2 using ECL Plus (Amersham Pharmacia, Piscataway,NJ) for visualization.

### Statistical Analysis

Quantitative data were expressed as the mean ± SD. A Student’s *t*-test was used to compare the measurement data of two groups. The reduction of rocuronium requirement in the anhepatic phase (relative to that in the paleohepatic phase) vs. serum bilirubin prior to the surgery was analyzed using a linear regression. Data from three or more samples were analyzed using one-way analysis of variance (ANOVA), followed by Dunnett’s t-test (the mean positivity of Oatps in tissue samples the Sham and 7, 14, 28-day BDL groups; the TOF50 time among blank, Oatp1-ShRNA and Oatp2-ShRNA groups) and Tukey’s multiple comparisons test (the TOF50 time from Sham + systemic metabolism, Sham + no-liver or no-kidney, BDL + systemic metabolism, BDL + no-liver or no-kidney; the excretion percentages of rocuronium though urine and bile). The level of statistical significance was set at *p* < *0.05.* All statistical analyses were performed with PASW Statistics version 18.

## Additional Information

**How to cite this article**: Wang, L. *et al*. Increased Renal Clearance of Rocuronium Compensates for Chronic Loss of Bile Excretion, via upregulation of Oatp2. *Sci. Rep.* **7**, 40438; doi: 10.1038/srep40438 (2017).

**Publisher's note:** Springer Nature remains neutral with regard to jurisdictional claims in published maps and institutional affiliations.

## Supplementary Material

Supplementary Figure S1

Supplementary Figure S2

Supplementary Figure S3

Supplementary Figure S4

## Figures and Tables

**Table 1:** Characteristics for the Children Who Underwent Orthotopic Liver Transplantation.

Children	Age (M)	Gender	Weight (kg)	Kasai Portoenterostomy	CTP	PT (s)	Total Bilirubin (μmol•L^−1^)	Albumin (g•L^−1^)	Ascites (ml)	Decreased Percentage of R* (%)
1	6.0	Female	6.5	no	11	16.0	615.8	27.1	mild	27
2	10	Female	8.0	yes	9	18.0	302.2	29.1	none	25
3	7.5	Female	8.0	no	11	20.9	578.2	34.7	none	38
4	6.0	Male	8.0	no	9	17.2	360.5	32	none	24
5	6.0	Female	8.3	no	10	14.0	247.1	25.8	mild	22
6	7.0	Male	6.8	yes	7	11.2	86.6	33.9	none	36
7	6.0	Female	6.5	yes	6	12.7	168	35.1	none	59
8	7.0	Male	6.0	yes	8	16.6	522.9	32.7	mild	6.8
9	10	Female	5.7	yes	7	9.9	176.5	39.4	none	18
10	8.0	Female	6.0	no	11	18.9	334.4	24.3	mild	33
11	12	Male	7.5	yes	6	18.9	21.0	35.7	none	43
12	12	Female	10	yes	6	15.5	86.3	45	none	39
13	7.0	Male	7.0	yes	8	11.0	293.4	31.8	none	43
14	6.0	Male	7.0	yes	8	12.0	294.1	31.2	none	33
15	6.0	Male	6.9	yes	6	13.1	109.9	35.4	none	39
16	6.0	Female	7.4	no	7	16.3	679.9	40	none	15

CTP = Child-Turcotte-Pugh.

*Reduction of rocuronium infusion requirements during the anhepatic phase relative to the palehepatic phase.
