# Supplementary material for: Increased Renal Clearance of Rocuronium Compensates for Chronic Loss of Bile Excretion, via upregulation of Oatp2
Source: Sci Rep. 2017 Jan 13;7:40438. doi: 10.1038/srep40438 (PMC5233986; doi:10.1038/srep40438)
Supplement: Supplementary Figure S2 [file srep40438-s2.pdf]

Increased Renal Clearance of Rocuronium Compensates for Chronic Loss of Bile Excretion, via upregulation of Oatp2

Authors: Long Wang, Mai-Tao Zhou, Wen Yin, Cai-Yang Chen, Chi-Wai Cheung, Li-Qun Yang, Wei-Feng Yu

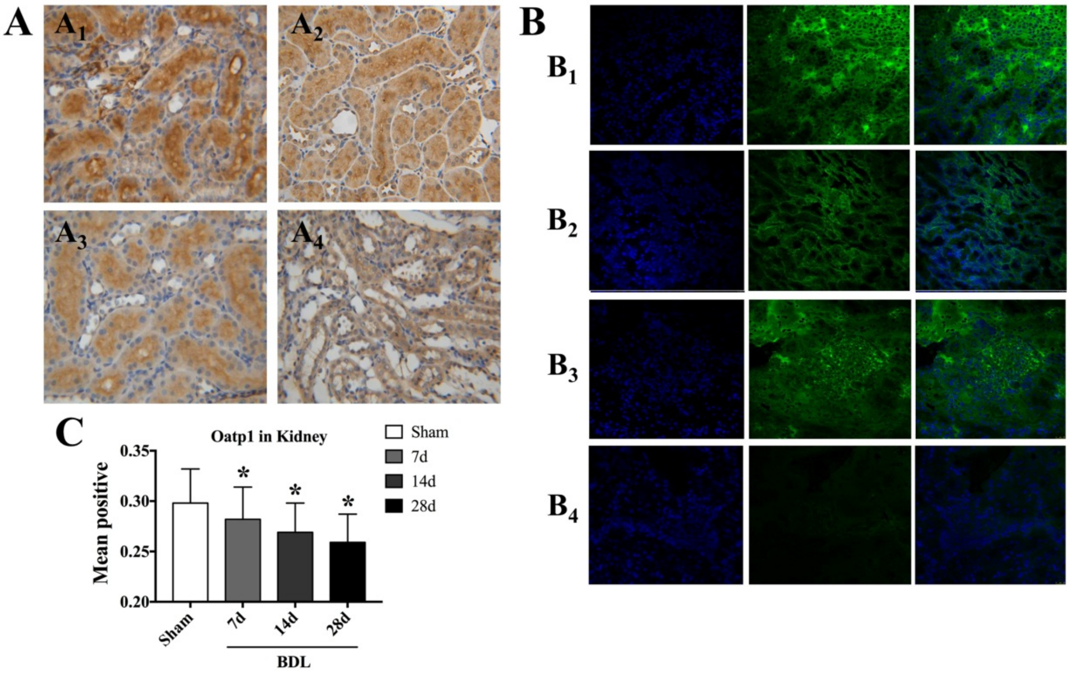

FigureS2. Renal expression of Oatp1 was down-regulated by BDL.

A represents expression of Oatp1 in Sham, 7d, 14d and 28d-BDL rats detected by IHC. **C** represents statistical analysis of positive Oatp1 in the four groups. **B:** Immunofluorescent for Oatp1 expression: B_1 sham rats; B_2 7d-BDL rats; B_3 14d-BDL rats; B_4 28d-BDL rats. *Significantly ($p< 0.05$) different from Sham.
