# Supplementary material for: Increased Renal Clearance of Rocuronium Compensates for Chronic Loss of Bile Excretion, via upregulation of Oatp2
Source: Sci Rep. 2017 Jan 13;7:40438. doi: 10.1038/srep40438 (PMC5233986; doi:10.1038/srep40438)
Supplement: Supplementary Figure S3 [file srep40438-s3.pdf]

Increased Renal Clearance of Rocuronium Compensates for Chronic Loss of Bile Excretion, via upregulation of Oatp2

Authors: Long Wang, Mai-Tao Zhou, Wen Yin, Cai-Yang Chen, Chi-Wai Cheung, Li-Qun Yang, Wei-Feng Yu

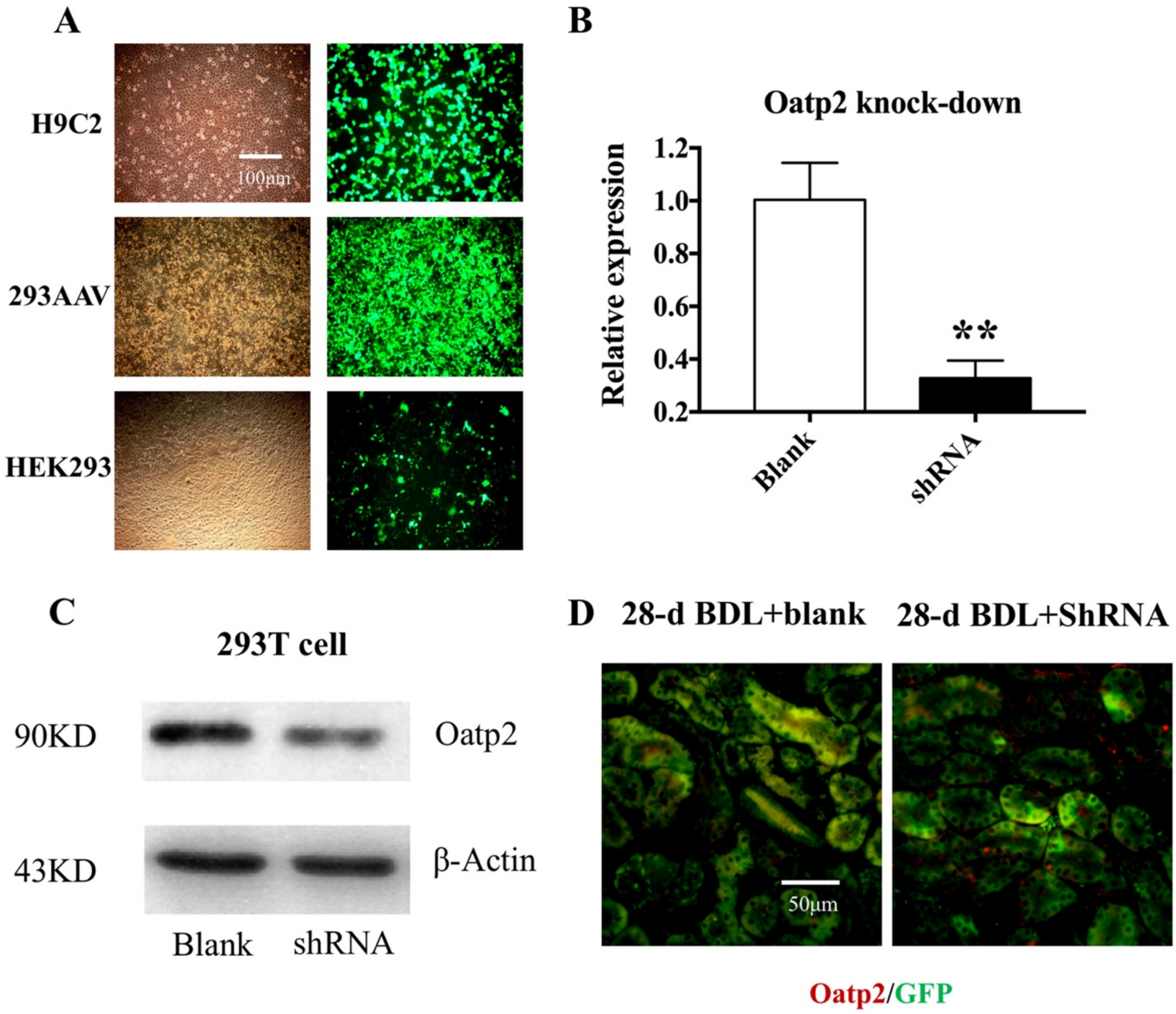

FigureS3. Efficiency of adeno-associated virus (AAV) vectors for gene transfer.

A: transfection efficiency in H9C2, 293AAV and HER293 cell line. **B:** qPCR of Oatp2 mRNA in blank and shRNA. **C:** western blot analysis of Oatp2 expression in blank and shRNA. The image has been cropped and 73KD represents Oatp2. **D:** Immunofluorescence of Oatp2 expression in blank and shRNA. Red signal represents Oatp2 protein, and green signal represents the protein of GFP embedded in AAV. **Significantly ($p < 0.01$) different from blank.
