# Supplementary material for: Increased Renal Clearance of Rocuronium Compensates for Chronic Loss of Bile Excretion, via upregulation of Oatp2
Source: Sci Rep. 2017 Jan 13;7:40438. doi: 10.1038/srep40438 (PMC5233986; doi:10.1038/srep40438)
Supplement: Supplementary Figure S4 [file srep40438-s4.pdf]

Increased Renal Clearance of Rocuronium Compensates for Chronic Loss of Bile Excretion, via upregulation of Oatp2

Authors: Long Wang, Mai-Tao Zhou, Wen Yin, Cai-Yang Chen, Chi-Wai Cheung, Li-Qun Yang, Wei-Feng Yu

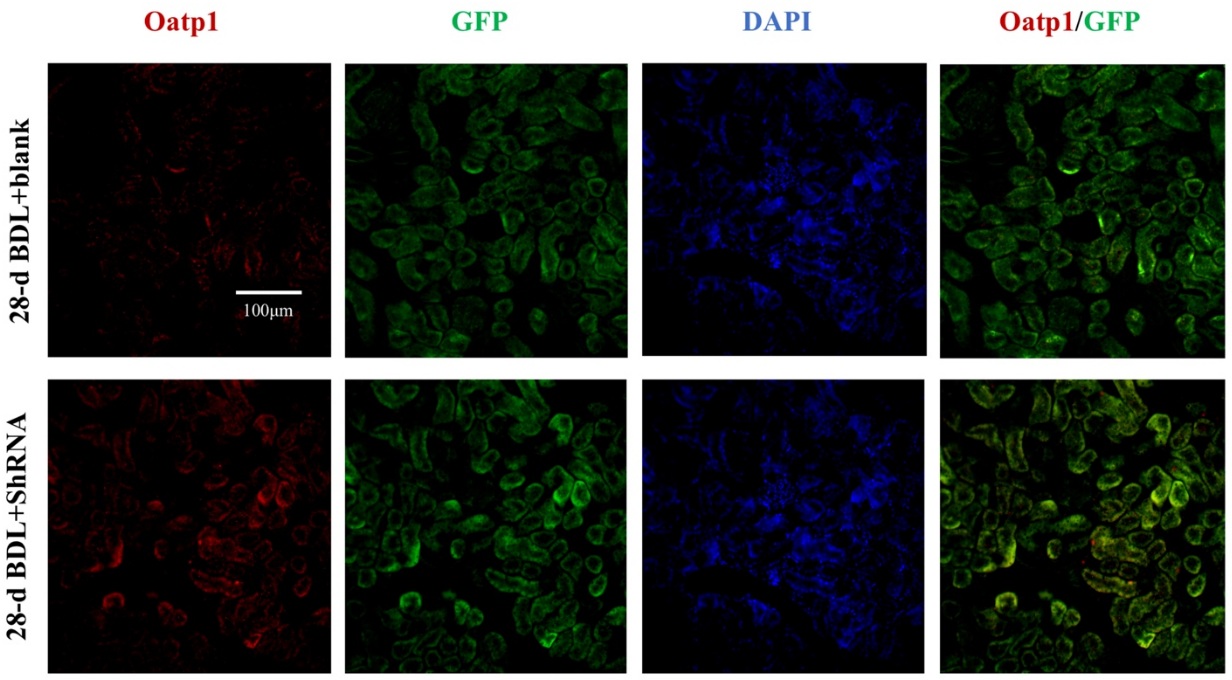

FigureS4. Immunofluorescence of Oatp1 expression in blank solution and AAV-rSlco1a1 administered groups.

Red signal represents Oatp1 protein, and green signal represents the protein of GFP embedded in AAV.
